# Reworking GWAS Data to Understand the Role of Nongenetic Factors in MS Etiopathogenesis

**DOI:** 10.3390/genes11010097

**Published:** 2020-01-14

**Authors:** Rosella Mechelli, Renato Umeton, Grazia Manfrè, Silvia Romano, Maria Chiara Buscarinu, Virginia Rinaldi, Gianmarco Bellucci, Rachele Bigi, Michela Ferraldeschi, Marco Salvetti, Giovanni Ristori

**Affiliations:** 1 IRCCS San Raffaele Pisana, San Raffaele Roma Open University, 00166 Rome, Italy; grazia.manfre@gmail.com; 2 Department of Informatics and Analytics, Dana-Farber Cancer Institute, Boston, MA 02215, USA; renato83@gmail.com; 3 Massachusetts Institute of Technology, Cambridge, MA 02139, USA; 4 Harvard T. H. Chan School of Public Health, Boston, MA 02115, USA; 5 Centre for Experimental Neurological Therapies (CENTERS), Department of Neurosciences, Mental Health and Sensory Organs, Sapienza University of Rome, 00189 Rome, Italy; silvia.romano@uniroma1.it (S.R.); mchiara.buscarinu@gmail.com (M.C.B.); virginia.rinaldi@mail.com (V.R.); bellucci.1638116@studenti.uniroma1.it (G.B.); rakele83@gmail.com (R.B.); michela.ferraldeschi@gmail.com (M.F.); giovanni.ristori@uniroma1.it (G.R.); 6 IRCCS Istituto Neurologico Mediterraneo (INM) Neuromed, 86077 Pozzilli, Italy

**Keywords:** genome wide association studies, multiple sclerosis, gene-environment interaction, expression quantitative trait loci (eQTL), protein–protein interaction, pathway analysis, polygenic risk score

## Abstract

Genome-wide association studies have identified more than 200 multiple sclerosis (MS)-associated loci across the human genome over the last decade, suggesting complexity in the disease etiology. This complexity poses at least two challenges: the definition of an etiological model including the impact of nongenetic factors, and the clinical translation of genomic data that may be drivers for new druggable targets. We reviewed studies dealing with single genes of interest, to understand how MS-associated single nucleotide polymorphism (SNP) variants affect the expression and the function of those genes. We then surveyed studies on the bioinformatic reworking of genome-wide association studies (GWAS) data, with aggregate analyses of many GWAS loci, each contributing with a small effect to the overall disease predisposition. These investigations uncovered new information, especially when combined with nongenetic factors having possible roles in the disease etiology. In this context, the interactome approach, defined as “modules of genes whose products are known to physically interact with environmental or human factors with plausible relevance for MS pathogenesis”, will be reported in detail. For a future perspective, a polygenic risk score, defined as a cumulative risk derived from aggregating the contributions of many DNA variants associated with a complex trait, may be integrated with data on environmental factors affecting the disease risk or protection.

## 1. Introduction

Multiple sclerosis (MS) is the most common chronic inflammatory disease of the central nervous system causing neurological disability. There is a strong need to understand the still elusive MS cause(s) and use this knowledge to develop safe drugs that specifically target disease mechanisms. Like other complex traits, MS results from the interaction of genetic and environmental factors [1,2]. While genetic studies emphasize the importance of polymorphisms in several genes regulating the immune response, the role of environmental factors is less clear.

Current multiple sclerosis (MS) research has been significantly affected by the increasing number of disease-modifying therapies (DMTs) that are now available for its relapsing-remitting phase. Nonetheless, some aspects remain the focus of considerable interest, e.g., the treatment of progressive MS, that is just coming out of its infancy [3]; the identification of a ‘therapeutic window’ to potentially treat the prodromal, reversible phase of the disease, a topic related to MS endophenotypes (coming from magnetic resonance imaging and/or predictive biomarkers; [4]); and an etiologic therapeutic approach, that is still out of reach, since molecular models and the complex interactions between the heritable, nonheritable, and stochastic events underlying the disease need to be detailed [5,6,7].

Contributions with respect to the last point may be made through genome-wide association studies (GWAS) that provide an unequaled tool by which to construct etiologic patterns, as well as to deepen our understanding of the complex interactions between heritable and nonheritable causal factors underpinning neuroinflammation. GWAS are population-based association studies, comparing disease cases and controls for common genetic variants at variable frequencies in the general population. They have identified more than 200 MS-associated loci across the human genome over the last decade [8,9,10,11]. Methodological advances, increased sample sizes, and improved bio-statistical approaches have all contributed to important progress in the definition of the genetic architecture of MS; complexity is now evident for disease genetics, which had, until 15 years ago, essentially been limited to the role of human leukocyte antigen (HLA). GWAS results are derived from each common variant (signaled by a single nucleotide polymorphism (SNP)) that explains a small fraction of the risk/protection in a given population. The overall genetic risk is largely due to many common variants of small effect spread throughout the genome, except for loci lying in the HLA complex and a handful of rare gene variants that have recently been associated with MS [12,13,14]. 

This complexity poses at least two challenges: i) understanding the plausible causal effects of the gene regions identified, as well as the impact of nongenetic factors, that, as demonstrated by the twin studies, is substantial and different in diverse populations [15,16]; and ii) translating genomic data in clinical practice through the repurposing of available drugs or the discovery of new druggable targets.

Concerning the first point, several works have dealt with single genes of interest, trying to understand whether and how MS-associated SNP variants affect the expression and the function of those genes. There are a few examples where, among the many signals resulting from GWAS, the causal variant and the underlying mechanism has been defined, such as the following. SNPs in the *interleukin 2 receptor* gene seem to differently influence various autoimmune diseases [17,18,19]. Likewise, variants of *TYK 2* locus resulting from GWAS in different immune-mediated conditions normally mediate a balanced genetic effect, warranting a trade-off between autoimmunity and immunodeficiency [20]. Next, SNPs in the *interleukin 7 receptor* gene affect MS genetic risk through an epistatic interaction, that, altering the regulation of exon 6 splicing, impacts on MS risk through its soluble form [21]. A brilliant example of how clinical practice can be informed by reworking GWAS data was given in a work on an MS-associated SNP in the *tumor necrosis factor (TNF) receptor* gene region, where the authors demonstrated that the genic variant mimicked the effect of TNF-blocking drugs in increasing the risk of demyelinating disease [22]. Finally, another recent finding (overexpression of the B cell-promoting cytokine BAFF due to an MS-associated genetic variant; [23]) seems to be in line with the substantial impact of B cell-depleting therapies on MS course [24], but is yet to be reconciled with the worsening effects resulting from an anti-BAFF trial on disease activity [24,25]. Overall, these studies have shown that GWAS reworking allows us to open some instructive windows on MS mechanisms, but they contribute only in part to the construction of a comprehensive etiologic model.

Concerning the clinical translation of genomic data with a focus on therapeutic targets, some attempts based on MS GWAS have emerged [26,27]. An interesting recent resource, reported by Fang and colleagues, developed a priority index (PI) pipeline, based on GWAS variants for several immunopathological traits, including MS [28]. By integrating functional genomic, immune-related annotations, and knowledge of network connectivity, the authors were able to maximize the genetic information for target validation of 30 immune traits. The added value of this approach was the incorporation of network connectivity information that increases enrichment for established therapeutic targets and helps overcome the possibility that many potential targets do not contain naturally-occurring variants that disrupt gene function and are associated with a relevant trait. A possible channel for the future development of similar approaches may lie in the bioinformatic reworking of GWAS data, considering the components of PI together with supposedly active, nonheritable factors which are known to interact with the genetic signals resulting from genome-scale data. 

## 2. Bioinformatic Reworking of GWAS Data

The analysis performed on GWAS data considered SNPs exceeding a *p*-value threshold of 5 × 10^−8^ as being relevant for association with the disease. This approach stems from the assumption that genetic markers independently contribute to disease development. Other approaches took into consideration a looser cutoff (*p*-values of less than 0.05), with the assumption that a combined effect of many loci, albeit with a modest contribution, may account for overall disease susceptibility (Table 1). 

Pathway analysis was one of the approaches selected for GWAS data interpretation, based on the identification of molecular function and biological processes that can be targeted by disease-associated SNPs [39]. An interpretation of nominally MS-associated SNPs (*p* < 0.05), obtained from two studies [30,31] and combining pathway analysis and protein–protein interaction networks (PINs), identified subnetworks of genes involved in several immunological and neural pathways which are enriched in MS [29]. In another study, this approach highlighted the oxidative stress and immune dysfunction pathways as being relevant in primary progressive MS, despite the fact that conventional GWAS analysis has not shown any association at the single SNP level [40]. To further unravel the heritable factors that are potentially involved in MS, a PIN-based pathway analysis (PINBPA, [41]) was conducted. Specifically, significant MS-associated SNPs (*p* < 0.05) of two independent GWAS datasets [8,33] were matched with data of protein–protein interaction networks that were subsequently gathered in a pathway analysis. This combined approach showed that proteins encoded by genes carrying risk variants are more likely to interact and share the same or related pathways. Moreover, the integration with protein–protein interaction pathways suggested new MS-susceptibility loci, including TNF-receptor-associated factor 3 (TRAF3), B cell membrane protein (CD48), B cell lymphoma 10 (BCL10), v-rel reticuloendotheliosis viral oncogene homolog (REL), and TEC protein tyrosine kinase (TEC) [32]. This approach proved to be informative. In fact, two *TRAF3* MS risk SNPs (rs12147246 and rs12588969; [11]) were then identified as being of genome-wide significance, while other studies have shown their involvement in a dysregulated response to EBV infection [42], i.e., one of the main environmental factors associated with MS [43,44]. 

Another approach of GWAS reworking is based on the wealth of information on regulatory elements which is available thanks to the efforts of Encyclopedia of DNA elements (ENCODE) and Regulome Epigenomics Consortiums [45,46]. These repositories can help to identify disease-associated functional variants that could have an impact on specific cell types. Epigenetic changes, including DNA methylation, posttranslational modification of histones, and the synthesis of noncoding RNAs, are representative of molecular mechanisms through which environmental signals are translated within the cells to change their gene expression [47,48]. In MS pathogenesis, a great deal of evidence suggests the integration of the risk related to genetic predisposition with cell-type-specific epigenetic changes occurring in the immune system and in the brain in response to environmental stimuli [49,50,51]. A reworking of GWAS data, aimed at identifying the cellular type where the MS-associated variants might exert functional effects, was recently performed in an association study that analyzed a total of 47,351 cases and 68,284 healthy controls [38]. Taking into consideration the 200 autosomal susceptibility variants outside the major histocompatibility complex (MHC), the authors considered all the regulatory elements that could be affected by the presence of these variants in a cell-specific manner, and created a cell-specific protein network. This paradigmatic approach to decoding the disease risk in a cell/tissue specific context suggested MS-associated variants operative in CNS resident microglia as important contributors (besides those of peripheral immune cells) to disease development [11,38].

A further level of complexity stems from the fact that many disease-associated polymorphisms identified by GWAS lie within regulatory regions of the genome. In fact, many MS-associated variants are located within noncoding regions [8,50] with a potential impact on gene expression as expression quantitative trait loci (eQTL). Currently available data on eQTL suggest a relationship between gene variants and the gene expression of certain cell types; these effects can be disease-specific, and possibly depend on external stimuli (i.e. signaling pathways activated by cytokines affecting a specific cell type) [50,52]. Specifically, most of eQTL have been studied in healthy volunteers, with the assumption that the effect of a single nucleotide variant may be independent from the diseased condition. Conversely, recent data has revealed a specificity of the eQTL effects in diseased subjects. A recent work based on RNA-Seq in PBMC from MS patients to identify eQTLs in regions centered on at-risk SNPs showed 77 statistically-relevant eQTL associations, 40% of which were more pronounced in MS patients compared with noninflammatory neurological disease patients [52]. Another interesting approach of eQTL analysis was based on public RNA-sequencing and microarray data of blood-derived cells. A group investigated the role of SNP rs1414273, which is located within the microRNA-548ac stem-loop sequence in the first intron of the CD58 gene. They provided evidence that this MS-associated SNP might alter Drosha cleavage activity, thus modifying CD58 and microRNA-548ac gene expression in immune cells, a change that has already been reported to be relevant for MS development [53,54,55]. 

## 3. Interactome-Based Approach

To understand a complex disease such as MS, the impact of environmental factors should be included in investigations. The influence of genetic variants on environmental exposures that associate with multifactorial diseases is far from being fully understood, particularly at a genome-wide level. The complexity of interactions between genes and environment needs to be explored using analytical approaches which are capable of considering many variables simultaneously; this may account for the so called ‘missing heritability’. New investigations on the aggregate analysis of many GWAS loci, each contributing with a small effect to overall disease predisposition, might uncover new information when combined with nongenetic factors that can play a role in disease etiology. We tried to implement this concept, using available actionable data on interactions at the protein level between human gene products and exposures. The possible causal significance of environmental exposures was investigated by measuring, at a genome-wide level, the enrichment of MS-associated genetic variants in genomic regions coding for proteins interacting with the exposure (i.e. influencing the exposure). Being aware that most SNPs associated with complex traits fall within regulatory genomic regions with distal effects or even in trans effects, we operationally chose to consider a 20kbp distance between MS variants and the nearest genes. This analysis was performed using Association LIst Go AnnoTatOR (ALIGATOR) bioinformatic tool [34] to search for statistical enrichment in associations between interactome’s genes and MS genome-wide association data published by IMSGC in 2011 [8]. 

**Figure 1:**
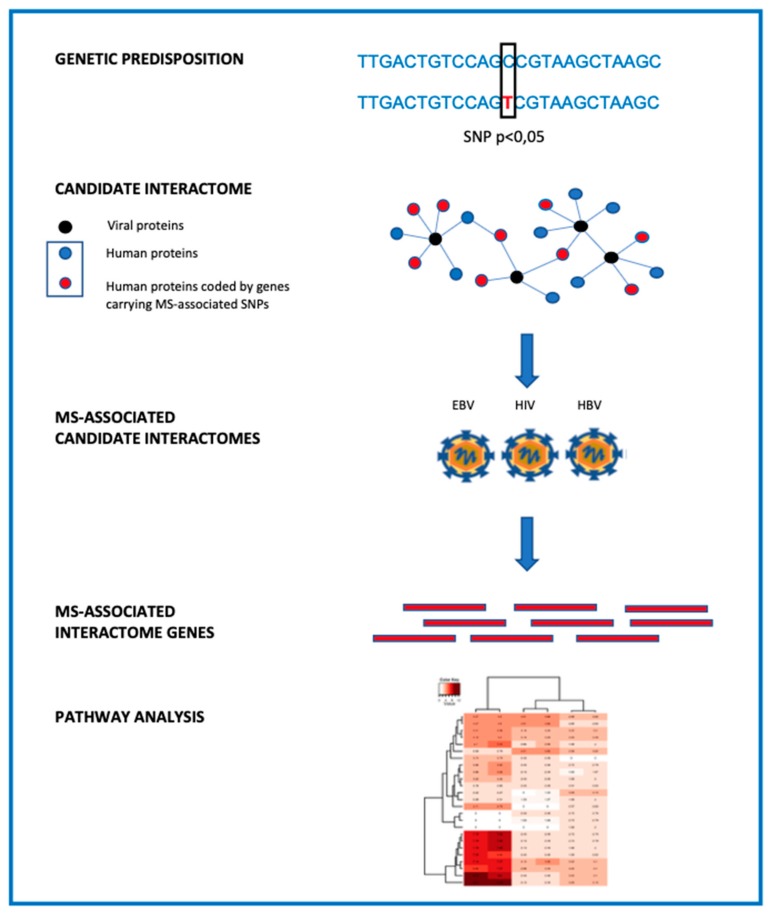
Schematic representation of the candidate-interactome approach.

The interactomes can be defined as “modules of genes whose products are known to physically interact with environmental or human factors with plausible, uncertain, or unlikely relevance for MS pathogenesis” [56]. The analysis was centered on viral interactomes, based on the classical hypothesis of a viral etiology of MS, examining only direct interactions between viral and human proteins, i.e., those of primary importance for the phenotypic impact of the environment within the host physiology [57,58]. This approach took into consideration MS-associated SNPs contributing with a small effect to overall disease susceptibility (*p*-value cut-off of association less than 0.05), thus starting from GWAS signal values which were well beyond those considered to be at the genome-wide significance cutoff. This candidate-interactome analysis allowed us to determine a relative low number of known or new nongenetic factors which are associated with MS risk/protection, and to formalize interplays between the heritable and nonheritable elements of possible causal nature. Specifically, the relevance and complexity of interactions between host genotype and EBV were disclosed, highlighting, through a pathway analysis, some cellular functions that may be affected by such interactions (Figure 1). Moreover, this approach has the power to identify other viruses, and related interacting proteins, which are potentially relevant for MS etiology [56]. 

Other complementary approaches may unveil the relevance of interactions between environmental factors and genetic predisposition. MS susceptibility regions may be preferentially targeted by both the viral and cellular proteins which are directly involved in molecular mechanisms that are able to translate environmental signals into cellular perturbations. Epstein-Barr nuclear 2 (EBNA2, a viral transactivator of viral and cellular genes) binding motifs were shown to be significantly enriched in genomic intervals associated with MS [35,37]. Another layer of complexity is represented by a striking overlap between MS-associated loci, EBNA2 binding motifs, and Vitamin D receptor binding sites [35], suggesting that a complex interplay between host genetic variants and known associated environmental factors [5] may contribute to disease development. These results indicate that looking at genetic predisposition through the lens of nonheritable risk factors represents an advancement in studies aiming at disclosing functional meaning and prioritizing genetic variants coming from GWAS data.

## 4. Future Perspectives

The above studies have highlighted the need to integrate different analytical approaches with the aim of deepening our understanding of MS pathophysiology and etiology. New approaches, still experimental, could have future applications in quantifying the overall burden of genetic risk factors or serving as a stratification biomarker for treatment optimization. A promising approach in this field is the polygenic risk score (PRS), also known as ‘genetic risk score’. PRS can be defined as a cumulative risk derived from aggregating the contributions of many DNA variants associated with a complex trait or disease. Existing research using PRS mainly focuses on two problems: association analysis and outcome prediction. 

Although the use of PRS has not yet achieved clinical accuracy levels, interesting potential perspectives have emerged in diseases like cancer [59], psoriasis [60], rheumatoid arthritis [61], mental disorders [62,63], atherosclerosis [64], Type 2 diabetes [65,66], asthma [67], Parkinson’s disease [68,69], and cardiovascular diseases (CVD) [70], including coronary heart disease (CHD) [71]. Polygenic risk scores can help to select a therapy for disease prevention. For example, statin therapy was shown to lead to a greater relative risk reduction for coronary heart disease among patients at high genetic risk score compared with patients at low genetic risk [63]. Polygenic risk scores have also been used to explore the genetic overlap between different diseases (e.g., application of schizophrenia-specific PRS to bipolar disorder), where the PRS derived from one disease is evaluated in another.

In a complex disease such as MS, the PRS could be correlated with phenotypic data (the ‘classical’ clinical and neurardiological parameters, but also to some emerging biomarkers that are being intensively investigated in biological fluids) as well as with MS endophenotypes (such as the radiologically-isolated syndrome; [72]) to generate a complex risk model which is able to predict the cumulative effects leading to overt disease onset. Moreover, the PRS may be integrated once again by data on environmental factors affecting disease risk or protection. In this context, it is possible to speculate on the calculation of an individual’s risk of disease based on the “candidate interactome” approach, integrating genetic-environmental interplay as reported in the previous paragraph. Also, the recently reported genomic variants of EBV [73,74], the main environmental risk factor for MS development, may prompt us to define a PRS combining the MS-associated loci of the host with the risky or protecting genomic variants of the virus. All these approaches would require a model that was calibrated to proportionately include genetic variants (G) and environmental exposures (E), i.e., taking into account G, E, and their interaction GxE. Such a tool does not currently seem to be within reach for complex diseases, where problems such as pleiotropy, causal effect estimation, and other questions of etiologic epidemiology remain. Overall, the etiology of multifactorial diseases seems to be more complex than anticipated [75]; therefore, their inherently multifaceted nature requires new models. Nonetheless, emerging bio-statistical approaches seem poised to start a post-GWAS era [76].

## Figures and Tables

**Table 1:** Main studies reworking genome-wide association studies (GWAS) data by considering multiple sclerosis (MS)-associated loci with different *p*-value cut-off.

Source	GWAS Datasets	*p*-Value Cut-Off of MS-Associated SNPs	Analysis	Results
Baranzini et al 2009 [29]	IMSGC, 2007 [30]; Baranzini et al. 2009 [31]	*p* < 0.05	Pathway analysis and protein–protein interaction (PPI)-network	Identification of immunological and neural pathways enriched in MS
IMSGC et al. 2013 [32]	IMSGC, 2011 [8]; Patsopoulos et al. 2011 [33]	*p* < 0.05	protein-interaction-network-based pathway analysis (PINBPA)	Identification of new MS susceptibility loci association blocks (groups of contiguous genes with a *p*-value < 0.05) including *BCL10, CD48, REL, TRAF3*, and *TEC*.
Mechelli et al. 2013 [34]	IMSGC, 2011 [8]	*p* < 0.05	Candidate-interactome approach	EBV is the most MS-associated environmental risk factor interacting with MS-associated SNPs.
Ricigliano et al. 2015 [35]	IMSGC 2013 [9]; Cree et al. 2010 [36]	*p* < 5 × 10^8^	Transcription factor binding	EBNA2 and VDR binding sites are enriched in MS-associated loci.
Harley et al 2018 [37]	NHGRI GWAS catalog	*p* < 5 × 10^8^	Transcription factor binding	EBNA2 binding sites are enriched in autoimmune-associated loci, including MS.
IMSGC et al. 2019 [38]	IMSGC 2019 [11]	*p* < 5 × 10^8^	Prioritization of cell specific gene/protein networks	Explanation of the potential role of GAWS signals in a tissue/cell-specific manner: identification of cell-specific susceptibility pathways.
IMSGC 2019 [11]	IMSGC 2019 [11]	*p* < 5 × 10^8^	Multiple approaches: cell-specific eQTL, pathway analyses; PPI	Prioritization of genes putatively associated with the disease, and identification of possible major implications for resident microglia and the B cell in MS.
